# The importance of Naples prognostic score in predicting long-term mortality in heart failure patients

**DOI:** 10.1080/07853890.2024.2442536

**Published:** 2024-12-14

**Authors:** Sidar Şiyar Aydın, Selim Aydemir, Murat Özmen, Emrah Aksakal, İbrahim Saraç, Faruk Aydınyılmaz, Onur Altınkaya, Oğuzhan Birdal, İbrahim Halil Tanboğa

**Affiliations:** a Department of Cardiology, Faculty of Medicine, Atatürk University, Erzurum, Turkey; b Department of Cardiology, University of Health Sciences, Erzurum City Hospital, Erzurum, Turkey; c Clinic of Cardiology, Hisar Intercontinental Hospital, Istanbul, Turkey

**Keywords:** Heart failure, inflammation, malnutrition, mortality, Naples prognostic score

## Abstract

**Background:**

Heart failure (HF) remains a significant health problem despite advances in diagnosis and treatment options. Malnutrition and increased inflammation predict poor disease prognosis. The parameters of the Naples prognostic score (NPS) include albumin, total cholesterol, neutrophil-lymphocyte ratio (NLR), and lymphocyte-monocyte ratio (LMR). We aimed to assess the potential of NPS as a predictor of long-term mortality in patients with HF.

**Methods:**

A total of 1728 patients with HF who applied to our center between 2018 and 2022 were included in this study. The NPS was computed and the patients were divided into three groups according to their NPS values as follows: NPS = 0 (Group 1), NPS = 1–2 (Group 2), and NPS = 3–4 (Group 3). We also evaluated the association between NPS value and HF mortality.

**Results:**

The patients were followed for a mean follow-up duration of 30 months. The mortality rate was 8.3% (145 patients). We carried out Model-1 and -2 Cox regression analyses to identify long-term mortality determinants. Model-2 was constructed by adding NPS to Model-1. NPS was significantly associated with HF mortality (Hazard Ratio: 2.194, 95% Confidence Interval: 1.176–4.091, *p* = 0.014). According to the Kaplan-Meier plot and log-rank analyses, there was a statistically significant difference in the long-term mortality of patients with HF and their NPS values for the entire cohort.

**Conclusion:**

Based on our findings, NPS showed promise as an independent predictor of long-term mortality in individuals with HF.

## Introduction

Heart failure (HF) continues to be a significant health problem despite advances in diagnosis and treatment options. Global epidemiological data have shown that HF affects ∼26 million people and has a 50% mortality rate within a decade [1,2]. The prevalence of HF is gradually increasing, especially due to the increased survival rate after acute cardiac events and the prolongation of average life expectancy [3]. Considering the increasing number of patients diagnosed with HF and the high treatment costs, tools that determine disease prognosis are still needed. Although many tools are used to predict HF prognosis, malnutrition, and increased inflammation also predict a poor prognosis of the disease [4,5]. The parameters of the Naples prognostic score (NPS) include albumin, total cholesterol, neutrophil-lymphocyte ratio (NLR), and lymphocyte-monocyte ratio (LMR). NPS can therefore indicate both inflammation and malnutrition status and has been used for the first time to predict the prognosis of patients undergoing surgery for colorectal malignancyI [6]. In subsequent studies, NPS was found to be associated with poor outcomes of some cardiovascular diseases, including HF with reduced ejection fraction (HFrEF) [7–9]. Based on ejection fraction determination, HF is divided into three groups: HFrEF, heart failure with mildly reduced ejection fraction (HFmrEF), and heart failure with preserved ejection fraction (HFpEF) [2]. To our knowledge, there have been no reports that probed the relationship between NPS and mortality in all three HF ejection fraction groups. For this reason, we aimed to assess the potential of NPS as a predictor of long-term mortality in patients of all three HF ejection fraction groups.

## Materials and methods

### Study design and patient selection

**Figure 1.**
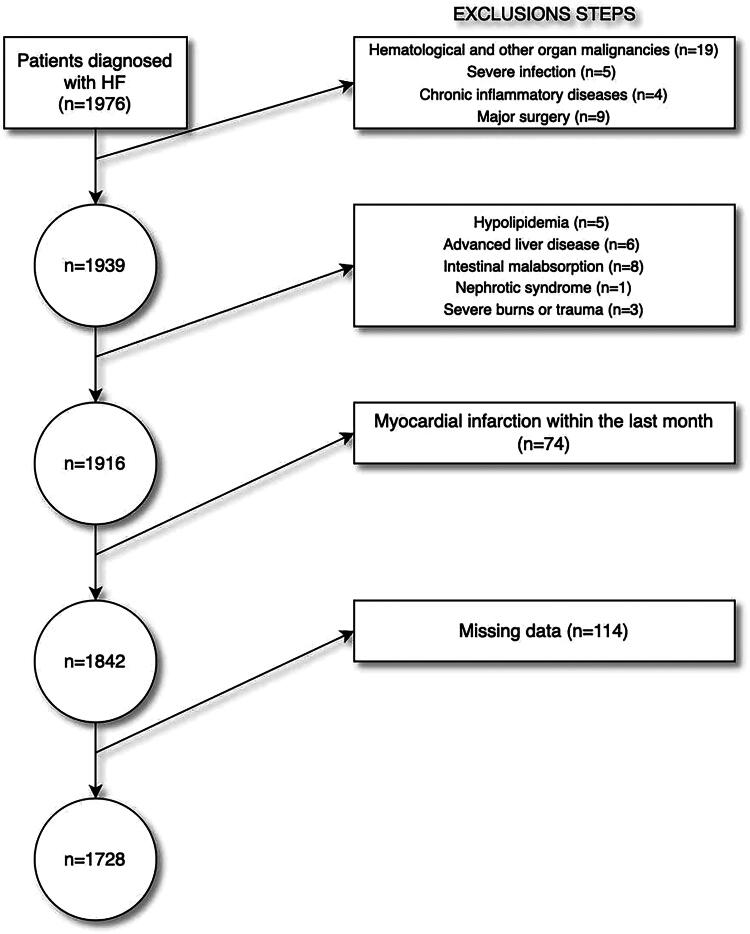
Flow chart of exclusion steps.

This was a single-center, retrospective study. Records of 1976 patients with HF who presented to our center between 2018 and 2022 were retrospectively extracted. Patients with hematological and other organ malignancies (*n* = 19), severe infection (*n* = 5), chronic inflammatory diseases (*n* = 4), major surgery (*n* = 9), hypolipidemia (*n* = 5), advanced liver disease (*n* = 6), intestinal malabsorption (*n* = 8), nephrotic syndrome (*n* = 1), severe burns or trauma (*n* = 3), myocardial infarction (*n* = 74) within the last month, and patients with missing data (*n* = 114) were excluded from the study (Figure 1). After exclusion, 1728 patients diagnosed with HF were included in the final analysis. The medical history of the patients, medication use history, clinical and demographic characteristics, hematological and biochemical parameters, echocardiographic parameters, and death information were obtained from electronic files. The NPS was calculated by using the albumin, total cholesterol, NLR, and LMR values at the time of application. Based on their NPS values, the patients were divided into three groups as follows: NPS = 0 (Group 1), NPS = 1–2 (Group 2), and NPS = 3–4 (Group 3). The relationship between NPS and HF mortality was also evaluated. The primary endpoint of the study was the mortality rate of the cohort. Written consent could not be obtained due to the retrospective design of this study. Verbal consent was obtained from the patients included in the study *via* telephone interviews. This study was performed in accordance with the Declaration of Helsinki and with the approval of the Erzurum City Hospital Ethical Committee, Turkey (Approval Number: 2024-01/01, Date: 10 January 2024).

### Definitions

The diagnosis of coronary artery disease (CAD) relied on invasive or non-invasive imaging techniques. The definition of diabetes mellitus (DM) was based on the guidelines of the American Diabetes Association [10]. Hypertension (HT) was defined as using any antihypertensive medication or systolic blood ­pressure >140 mmHg and/or diastolic blood pressure >90 mmHg [11].

Patients with symptoms of HF, such as shortness of breath, ankle swelling, and fatigue, in addition to signs, such as elevated jugular venous pressure, pulmonary crackles, and peripheral edema, were categorized based on their left ventricular ejection fraction (LVEF) measurements: those with LVEF ≤40% were identified as having HFrEF, individuals with LVEF falling between 41 and 49% were classified under HFmrEF, and patients with LVEF ≥50% were deemed to have HFpEF. The modified Simpson method was used to calculate LVEF [12]. NPS accrued 1 point if total cholesterol (TC) was <180 mg/dL, 1 point if albumin <40 g/L, 1 point if NLR >2.96, and 1 point if LMR ≤4.44. Otherwise, 0 points were added for each parameter [6].

### Statistical analysis

Continuous variables are presented as medians and interquartile ranges, and categorical variables are expressed as numbers and percentages. Baseline and clinical characteristics were assessed based on mortality status and NPS value using the Kruskal-Wallis and Chi-square tests.

Statistical modeling was carried out using multivariate Cox proportional hazards regression analysis. First, a base model (Model-1) was created using age, sex, HT, DM, CAD, LVEF, hemoglobin, chronic obstructive pulmonary disease (COPD), C-reactive protein (CRP), gamma-glutamyl transferase (GGT), creatine, and potassium. Model 2 was created by adding NPS to the model. The predictive accuracy and discriminative ability of these models were evaluated using the area under the curve (AUC) and *R*^2^. The association between all-cause mortality and NPS was measured using hazard ratio (HR) and 95% confidence interval (CI) values. Statistical significance was set at *p* < 0.05. All statistical analyses were performed using R studio software version 3.6.3 (R Project, Vienna, Austria) with the ‘rms’ package.

## Results

Our study cohort comprised 1728 patients who presented to our center for HF treatment between 2018 and 2022. The mean follow-up period was 30 months. We observed a mortality rate of 8.3% (145 patients). The mean age of the patients who died was 77 years, and that of the survivors was 70 years (*p* < 0.001). Forty percent of the patients who died (*n* = 58) and 49% of the survivors (*n* = 780) were men (*p* = 0.032). White cell blood (WBC), neutrophil, CRP, NLR, aspartate aminotransferase, alanine aminotransferase, GGT, creatine, sodium, potassium, and glucose levels were significantly higher in the deceased patient group. Hemoglobin, platelet count, LMR, TC, and albumin levels were significantly lower in the same group. Among the patients with a death outcome, the NPS distribution (Table 1) was NPS = 0 in 2.1% (3 patients), NPS = 1–2 in 46% (66 patients), and NPS = 3–4 in 52% (76 patients).

**Table 1.** Basal characteristics.

Variables	Mortality (−) (*n* = 1583)	Mortality (+) (*n* = 145)	*p*-Value
Age (years)	70 (61–78)	77 (68–85)	**<0.001**
Gender (male) (*n*, %)	780 (49)	58 (40)	**0.032**
CVE (*n*, %)	71 (4.5)	10 (6.9)	0.189
CAD (*n*, %)	1067 (67)	86 (59)	**0.048**
HT (*n*, %)	1322 (84)	104 (72)	**<0.001**
DM (*n*, %)	369 (23)	36 (25)	0.680
COPD (*n*, %)	338 (21)	55 (38)	**<0.001**
AF (*n*, %)	618 (39)	55 (38)	0.793
LVEF (%)	45 (35–55)	45 (35–55)	0.270
WBC (10^3^/µL)	8.3 (6.6–10.4)	10.2 (7.8, 14.6)	**<0.001**
Hemoglobin (g/dL)	14.2 (12.7–15.6)	12.9 (11.0–14.6)	**<0.001**
Eosinophil (10^3^/µL)	0.13 (0.07–0.23)	0.11 (0.05–0.26)	0.310
Monocyte (10^3^/µL)	0.83 (0.68–1.02)	0.89 (0.69–1.08)	0.128
Basophil (10^3^/µL)	0.5 (0.3–0.7)	0.5 (0.2–0.7)	0.792
Neutrophil (10^3^/µL)	6.7 (5.9–7.7)	7.5 (6.3–8.5)	**<0.001**
Lymphocyte (10^3^/µL)	2.4 (1.7–3.0)	1.8 (1.1–2.6)	**<0.001**
Platelet (10^3^/µL)	241 (198–292)	232 (173–320)	0.614
CRP (mg/L)	7 (2–25)	28 (10–73)	**<0.001**
NLR	2.9 (2.0–4.5)	4.3 (2.6–7.9)	**<0.001**
LMR	2.75 (1.90–3.78)	2.03 (1.34–2.90)	**<0.001**
Albumin (g/L)	41 (36–44)	37 (33–41)	**<0.001**
AST (mg/dL)	25 (19–36)	31 (21–77)	**<0.001**
ALT (mg/dL)	21 (15–32)	22 (17–48)	**0.004**
GGT (mg/dL)	18 (0–36)	32 (13–67)	**<0.001**
Glucose (mg/dl)	120 (97–169)	145 (111–199)	**<0.001**
TC (mg/dl)	163 (133–192)	147 (116–188)	**0.001**
LDL-C (mg/dl)	107 (81–134)	97 (68–139)	0.128
Triglyceride (mg/dl)	126 (93–174)	113 (85–170)	0.065
Creatinine (mg/dl)	0.99 (0.79–1.23)	1.33 (0.92–2.03)	**<0.001**
Sodium (mmol/L)	140 (137–142)	141 (138–144)	**0.003**
Potassium (mmol/L)	4.33 (3.99–4.73)	4.48 (4.04–5.18)	**0.002**
OAC (*n*, %)	458 (29)	47 (32)	0.378
Statin (*n*, %)	386 (24)	34 (23)	0.801
Ranolazine (*n*, %)	86 (5.4)	4 (2.8)	0.165
Carvedilol (*n*, %)	261 (16)	26 (18)	0.655
Ivabradine (*n*, %)	212 (13)	8 (5.5)	**0.006**
Metoprolol (*n*, %)	767 (48)	73 (50)	0.663
Antiplatelet agents (*n*, %)	780 (49)	66 (46)	0.386
NPS (*n*, %)			
0	106 (6.7)	3 (2.1)	
1–2	946 (60)	66 (46)	**<0.001**
3–4	531 (34)	76 (52)	

CVE: cerebrovascular event; CAD: coronary artery disease; HT: hypertension; DM: diabetes mellitus; COPD: chronic obstructive pulmonary disease; AF: atrial fibrillation; LVEF: left ventricular ejection fraction; WBC: white blood cell; CRP: c-reactive protein; NLR: neutrophil lymphocyte ratio; LMR: lymphocyte monocyte ratio; AST: acetate aminotransferase; ALT: alanin aminotransferase; GGT: gama-glutamyl transferase; TC: total cholesterol; LDL-C: low-density lipoprotein cholesterol; OAC: oral anticoagulans; NPS: naples prognostic score.

Based on the NPS values of our study cohort, the patients were divided into three groups as follows: NPS = 0 (Group 1), NPS = 1–2 (Group 2), and NPS = 3–4 (Group 3). Group 1 comprised 6.3% (109 patients), Group 2 58.5% (1012 patients), and Group 3 35.1% (607 patients) of the entire population. The mean age was 63 years in Group 1, 70 years in Group 2, and 73 years in Group 3; the difference was statistically significant (*p* < 0.001). The LVEF was 50% in group 1 and 45% in groups 2 and 3, with no significant difference. The WBC count was the highest in group 3 and was statistically significant (*p* < 0.001). The CRP level and NLR were significantly higher in Group 3 than in the other groups (*p* < 0.001 and *p* < 0.001, respectively). The LMR and albumin, TC, low-density lipoprotein, and triglyceride levels were significantly lower in Group 3 than in the other groups (*p* < 0.001, *p* < 0.001, *p* < 0.001, and *p* < 0.001, respectively). Aspartate aminotransferase, GGT, glucose, and creatinine levels were the highest in Group 3 and were statistically significant (*p* < 0.001, *p* < 0.001, and *p* < 0.001, respectively). The mortality rate was 2.8% (three patients) in Group 1, 6.5% (66 patients) in Group 2, and 13% (76 patients) in Group 3, and a statistically significant difference was detected (*p* < 0.001) (Table 2).

**Table 2.** General population according to discriminations of Naples prognostic score.

Variables	NPS = 0 (*n* = 109)	NPS = 1–2 (*n* = 1012)	NPS = 3–4 (*n* = 607)	*p*-Value
Age (years)	63 (55–74)	70 (61–77)	73 (65–80)	**<0.001**
Gender (male) (*n*, %)	51 (47)	498 (49)	289 (48)	0.770
CVE (*n*, %)	2 (1.8)	40 (4.0)	39 (6.4)	**0.026**
CAD (*n*, %)	79 (72)	710 (70)	364 (60)	**<0.001**
HT (*n*, %)	93 (85)	845 (83)	488 (80)	0.206
DM (*n*, %)	28 (26)	263 (26)	114 (19)	**0.003**
COPD (*n*, %)	14 (13)	203 (20)	176 (29)	**<0.001**
AF (*n*, %)	33 (30)	377 (37)	263 (43)	**0.008**
LVEF (%)	50 (35–60)	45 (35–55)	45 (30–55)	0.161
WBC (10^3^/µL)	7.7 (6.4–9.6)	8.1 (6.4–10.2)	9.2 (7.3–12.1)	**<0.001**
Hemoglobin (g/dL)	15.1 (14.1–16.1)	14.3 (12.8–15.5)	13.5 (12.0–15.2)	**<0.001**
Eosinophil (10^3^/µL)	0.16 (0.11–0.25)	0.15 (0.08–0.24)	0.11 (0.04–0.21)	**<0.001**
Monocyte (10^3^/µL)	0.66 (0.58–0.75)	0.87 (0.71–1.06)	0.81 (0.66–1.00)	**<0.001**
Basophil (10^3^/µL)	0.60 (0.40–0.80)	0.50 (0.30–0.80)	0.40 (0.20–0.60)	**<0.001**
Neutrophil (10^3^/µL)	5.3 (4.9–5.9)	6.3 (5.7–7.2)	7.7 (7.0–8.5)	**<0.001**
Lymphocyte (10^3^/µL)	3.8 (3.3–4.2)	2.7 (2.2–3.2)	1.6 (1.1–2.0)	**<0.001**
Platelet (10^3^/µL)	237 (198–294)	243 (198–292)	235 (191–295)	0.719
CRP (mg/L)	4 (1–7)	6 (1–21)	14 (4–41)	**<0.001**
NLR	1.4 (1.2–1.8)	2.4 (1.8–3.1)	4.8 (3.6–7.3)	**<0.001**
LMR	5.57 (5.07–6.27)	3.08 (2.35–3.92)	1.87 (1.31–2.53)	**<0.001**
Albumin (g/L)	43 (40–45)	41 (37–44)	39 (33–43)	**<0.001**
AST (mg/dL)	24 (18–28)	24 (18–36)	28 (20–43)	**<0.001**
ALT (mg/dL)	22 (16–31)	21 (15–31)	21 (16–36)	0.144
GGT (mg/dL)	14 (0–28)	16 (0–33)	23 (10–49)	**<0.001**
Glucose (mg/dl)	106 (94–148)	117 (96–164)	129 (105–184)	**<0.001**
TC (mg/dl)	209 (192–238)	175 (145–207)	139 (118–157)	**<0.001**
LDL-C (mg/dl)	144 (125–167)	113 (87–145)	85 (65–110)	**<0.001**
Triglycerides (mg/dl)	187 (145–274)	137 (99–189)	108 (82–141)	**<0.001**
Creatinine (mg/dl)	0.96 (0.75–1.11)	0.99 (0.81–1.22)	1.05 (0.82–1.39)	**<0.001**
Sodium (mmol/L)	140 (138–142)	140 (138–142)	140 (137–142)	0.234
Potassium (mmol/L)	4.33 (4.05–4.72)	4.33 (4.01–4.72)	4.36 (3.95–4.82)	0.613
OAC (*n*, %)	25 (23)	271 (27)	209 (34)	**0.002**
Statin (*n*, %)	27 (25)	260 (26)	133 (22)	0.227
Ranolazin (*n*, %)	6 (5.5)	63 (6.2)	21 (3.5)	0.052
Carvedilol (*n*, %)	19 (17)	167 (17)	101 (17)	0.969
Ivabradine (*n*, %)	22 (20)	134 (13)	64 (11)	**0.016**
Metoprolol (*n*, %)	50 (46)	504 (50)	286 (47)	0.486
Antiplatelet agents (*n*, %)	59 (54)	523 (52)	264 (43)	**0.003**
Mortality (*n*, %)	3 (2.8)	66 (6.5)	76 (13)	**<0.001**

CVE: cerebrovascular event; CAD: coronary artery disease; HT: hypertension; DM: diabetes mellitus; COPD: chronic obstructive pulmonary disease; AF: atrial fibrillation; LVEF: left ventricular ejection fraction; WBC: white blood cell; CRP: c-reactive protein; NLR: neutrophil lymphocyte ratio; LMR: lymphocyte monocyte ratio; AST: acetate aminotransferase; ALT: alanin aminotransferase; GGT: gama-glutamyl transferase; TC: total cholesterol; LDL-C: low-density lipoprotein cholesterol; OAC: oral anticoagulans.

**Figure 2.**
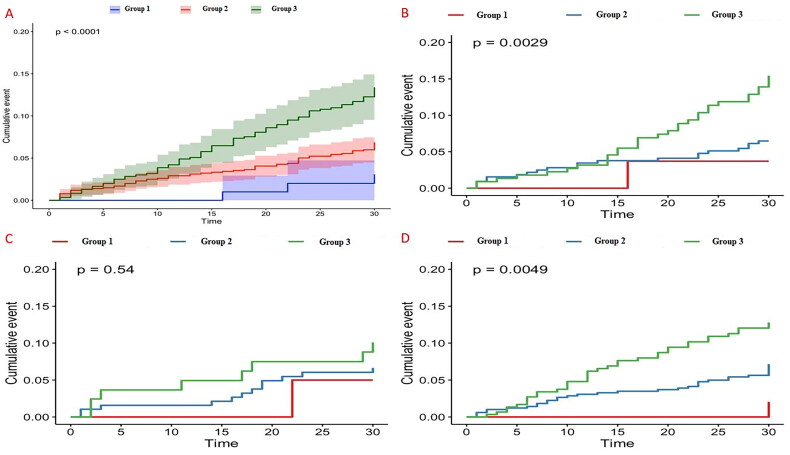
Kaplan Meier survival analysis of the entire population and subgroups (A: Whole population, B: Patients with HFrEF, C: Patients with HFmrEF, D: Patients with HFpEF).

According to the Kaplan-Meier plot and log-rank analysis, there was a statistically significant difference in the long-term HF mortality rates between the various NPS scores in the entire study cohort. Moreover, although this difference was statistically significant in both the patients with HFrEF and HfpEF, there was no statistically significant difference in the patients with HFmrEF (Figure 2). Model-1 and -2 were constructed to identify the determinants of long-term mortality in patients with HF and demonstrate the importance of NPS as a prognostic indicator. Model-2 was constructed by adding NPS to Model-1. A thorough analysis was conducted to compare both models. When NPS was added to Model-1, the time-dependent AUC was used as a distinctive feature of the improvements in the model, and *R*^2^ was used for predictive performance. With the addition of NPS to Model-1, a statistically significant increase was observed in the predictive performance of Model-2 (likelihood ratio *X*^2^ *p*-value: 0.012). NPS was significantly associated with HF mortality (HR: 2.194, 95% CI: 1.176–4.091, *p* = 0.014). The results of Cox regression analyses for Model-1 and -2 are presented in Table 3.

**Table 3.** Cox regression analyses of models for predicting mortality.

Variables	Model-1 (HR, 95% CI)	*p*-Value	Model-2 (HR, 95% CI)	*p*-Value
Age	2.05 (1.59–2.65)	**<0.001**	1.96 (1.52–2.54)	**<0.001**
Gender (male)	0.64 (0.45–0.91)	**0.014**	0.65 (0.46–0.92)	**0.015**
HT	0.58 (0.40–0.86)	**0.007**	0.60 (0.41–0.89)	**0.01**
DM	1.45 (0.98–2.14)	0.058	1.48 (1.01–2.18)	**0.047**
CAD	0.89 (0.61–1.28)	0.540	0.93 (0.65–1.34)	0.704
LVEF	0.93 (0.73–1.20)	0.610	0.95 (0.74–1.22)	0.715
Hemoglobin	0.98 (0.88–1.09)	0.767	0.99 (0.90–1.09)	0.856
COPD	1.68 (1.18–2.37)	**0.003**	1.64 (1.16–2.32)	**0.005**
CRP	1.17 (1.10–1.25)	**<0.001**	1.17 (1.09–1.24)	**<0.001**
GGT	1.11 (1.04–1.18)	**0.001**	1.11 (1.04–1.18)	**0.001**
Creatinine	0.99 (0.98–1.01)	0.807	1.00 (0.99–1.01)	0.769
Potassium	0.99 (0.96–1.02)	0.869	1.00 (0.97–1.02)	0.888
NPS	–	–	2.19 (1.17–4.09)	**0.014**
AUC	0.735	–	0.743	–
R^2^	0.092	–	0.097	–

HR: hazard ratio; CI: confidence intervals; HT: hypertension; DM: diabetes mellitus; CAD: coronary artery disease; LVEF: left ventricular ejection fraction; COPD: chronic obstructive pulmonary disease; CRP: c-reactive protein; GGT: gama-glutamyl transferase; NPS: naples prognostic score; AUC: area under the curve.

## Discussion

Various factors affect HF mortality, including laboratory parameters, clinical features, comorbid conditions, and treatment strategies. This study emphasized the potential of NPS to predict mortality in patients with HF. In predicting mortality, NPS showed better results compared to other HF mortality indicators like HT, DM, age, CAD, low hemoglobin levels, and high CRP levels. To our knowledge, this study is the first to explore the association between NPS and HF mortality, taking into account patients with HFrEF, HFmrEF, and HfpEF.

NPS includes parameters that evaluate malnutrition and inflammation. A meta-analysis conducted to assess malnutrition found that albumin and TC were significantly lower in both acute and chronic malnutrition patients [13]. These malnutrition markers (albumin, TC) can be commonly observed in patients with HF. Congestive enteropathy, which may occur as a result of HF, may be a cause of anorexia and malnutrition. In addition, chronic inflammation occurring during HF may cause cardiac cachexia [14,15]. Atrophy and fibrosis in cardiac myocytes, which may occur as a result of malnutrition, can cause the HF condition to worsen [16]. Additionally, one study found malnutrition to be closely associated with HF mortality [17]. Similar to previous reports, our study also found lower levels of albumin and TC, which are known indicators of malnutrition, in the deceased patient group of our study cohort.

Inflammation negatively affects disease severity and prognosis in patients with HF [18]. In particular, the WBC count and its subparameters were found to be closely associated with both hospitalization and long-term mortality. While high neutrophils numbers seen after myocardial infarction may be an indicator of acute HF, it has been suggested that there is a connection between low lymphocytes and HF mortality [19,20]. NLR is a new and popular biomarker obtained by measuring the ratio of neutrophils and lymphocytes. In the literature, NLR has been reported to be a predictor of HF mortality and other cardiovascular diseases [21,22]. Similarly, the group that experienced mortality in our study had a higher NLR.

Notwithstanding the specific cause of HF, ventricular remodeling and fibrosis play a crucial role in its prognosis. Cytokines released from blood cells, such as lymphocytes, granulocytes, and monocytes can cause inflammation in the heart and trigger remodeling and fibrosis. The organismal response to stress can trigger a decline in lymphocytes, while an elevation in monocytes is frequently associated with chronic inflammation [23,24]. LMR is determined by the ratio of lymphocyte to monocyte. A recent study found higher mortality rates in patients with HF exhibiting low LMR values [25]. Similarly, in our study, LMR values were lower in the group with a death outcome.

Since NPS includes parameters that reflect the inflammatory process and malnutrition, it can be a helpful tool in the prognosis of a chronic disease, such as HF. Recent studies have shown a link between NPS and HF mortality. In these studies, it was observed that HF mortality increased as NPS value increased. One of these studies included only HFrEF patients [9]. The other study included HFrEF and HFmrEF patients [26]. Our study differs from these reports in several aspects. To our knowledge, this is the first study to incorporate NPS values of patients with HFrEF, HFmrEF, and HFpEF. Additionally, unlike previously published studies, it has a larger sample size and an extended follow-up period.

## Conclusion

Based on our findings, NPS shows promise as an independent predictor of long-term mortality in individuals with HF. High NPS levels were associated with higher mortality rates, particularly in patients with HFrEF and HFpEF. NPS is a scoring system that is easy to calculate and is a low-cost option due to the parameters it includes. It should be kept in mind that high NPS values may be associated with poor prognosis, especially in patients with HF whose clinical course is considered critical.

## Limitations

This was a retrospective study. Hence, the limitations of our study included missing data, such as number of patients receiving optimal treatment recommended by the current guidelines, the functional capacity of the patients, and the circulating levels of natriuretic peptide.

## Data Availability

The datasets generated for this study are available on request to the corresponding author.
